# Association of exercise training and angiotensin-converting enzyme 2 activator improves baroreflex sensitivity of spontaneously hypertensive rats

**DOI:** 10.1590/1414-431X20165349

**Published:** 2016-08-01

**Authors:** P.R. Lopes, M.C.S. Moreira, S.M. Marques, I.S.J. Pinto, L.M. Macedo, C.C. Silva, A.H. Freiria-Oliveira, A.C.S. Rebelo, A.A.S. Reis, D.A. Rosa, M.L. Ferreira-Neto, C.H. Castro, G.R. Pedrino

**Affiliations:** 1 Centro de Pesquisas em Neurociência e Fisiologia Cardiovascular, Departamento de Ciências Fisiológicas, Instituto de Ciências Biológicas, Universidade Federal de Goiás, Goiânia, GO, Brasil; 2 Departamento de Ciências Fisiológicas, Instituto de Ciências Biológicas, Universidade Federal de Goiás, Goiânia, GO, Brasil; 3 Departamento de Morfologia, Instituto de Ciências Biológicas, Universidade Federal de Goiás, Goiânia, GO, Brasil; 4 Departamento de Bioquímica e Biologia Molecular, Instituto de Ciências Biológicas, Universidade Federal de Goiás, Goiânia, GO, Brasil; 5 Faculdade de Educação Física, Universidade Federal de Uberlândia, Uberlândia, MG, Brasil

**Keywords:** Intrinsic heart rate, Diminazene aceturate, Aerobic training, Double autonomic blockade, Renin-angiotensin system

## Abstract

The present study sought to determine cardiovascular effects of aerobic training associated with diminazene aceturate (DIZE), an activator of the angiotensin converting enzyme 2, in spontaneously hypertensive rats (SHRs). Male SHRs (280–350 g) were either subjected to exercise training or not (sedentary group). The trained group was subjected to 8 weeks of aerobic training on a treadmill (five times a week, lasting 60 min at an intensity of 50–60% of maximum aerobic speed). In the last 15 days of the experimental protocol, these groups were redistributed into four groups: i) sedentary SHRs with daily treatment of 1 mg/kg DIZE (S+D1); ii) trained SHRs with daily treatment of 1 mg/kg DIZE (T+D1); iii) sedentary SHRs with daily treatment of vehicle (S+V); and iv) trained SHRs with daily treatment of vehicle (T+V). After treatment, SHRs were anesthetized and subjected to artery and femoral vein cannulation prior to the implantation of ECG electrode. After 24 h, mean arterial pressure (MAP) and heart rate (HR) were recorded; the baroreflex sensitivity and the effect of double autonomic blockade (DAB) were evaluated in non-anesthetized SHRs. DIZE treatment improved baroreflex sensitivity in the T+D1 group as compared with the T+V and S+D1 groups. The intrinsic heart rate (IHR) and MAP were reduced in T+D1 group as compared with T+V and S+D1 groups. Hence, we conclude that the association of exercise training with DIZE treatment improved baroreflex function and cardiovascular regulation.

## Introduction

Aerobic exercise has been reported as a non-pharmacological antihypertensive therapy (1–5). Although physical exercise is known to induce autonomic and hemodynamic changes in hypertensive patients and spontaneously hypertensive rats (SHRs), some of the mechanisms that underlie reduction in arterial blood pressure (BP) are still unclear (2–6). Some studies have shown that aerobic exercise could promote a decrease in sympathetic activity (5), a physiological hypertrophy of the left ventricle (1) and improve the vascular reactivity (1,2). Although, some studies have suggested the existence of a relationship between exercise and renin-angiotensin system (RAS), more studies are needed to verify the role of RAS (4,7).

There is growing evidence in support of the relationship between high BP and cardiovascular complications (2). In clinical practice, high BP and decreased baroreflex sensitivity are indicators of hypertension (5,8,9). Interestingly, the oscillation in BP is directly related to organ damage (especially kidneys and hearts) (8). A decrease in baroreflex sensitivity has been reported in patients with post-myocardial infarction (10) and renal failure (11). The integrity of baroreflex sensitivity is important in the preservation and maintenance of cardiovascular homeostasis.

The RAS plays important roles in the regulation of systemic BP, fluid and electrolyte balance (7). This system is activated through the conversion of angiotensinogen to angiotensin (Ang I) by renin (12). Ang I is further cleaved by the angiotensin-converting enzyme (ACE) to release Ang II (12). Ang II is a potent vasoconstrictor that is responsible for the homeostatic maintenance of the cardiovascular system. Other important peptides that are released from RAS are Ang-(1-7) (3), which have many beneficial activities including vasodilatation, antifibrosis and antihypertrophic effects (3,4,12). This peptide is produced mainly through the action of ACE2, an ACE-homologous enzyme (13,14).

Previous reports have shown that the development and maintenance of hypertensive state in SHRs is associated with an increase in sympathetic nerve activity, left ventricular diastolic dysfunction and increase in plasma Ang II vasoconstrictor axis (Ang II-AT1) (15–19). The effects of training exercise on RAS have been the focus of several studies. Recently, the RAS vasoconstrictor axis downregulation in the renal artery (without affecting the vasodilator axis) was observed with aerobic exercise (20).

Previous studies have shown that moderate training exercise in combination with Ang-(1-7) prevents cardiac remodeling in the early course of hypertension in 2K1C rats (4). In addition, oral administration of HPB-CD/Ang-(1-7) has beneficial effects similar to physical exercise in SHRs (3). ACE2 activity exerts a negative regulation of RAS by opposing the role of ACE/Ang II/AT1 receptor axis (14). Díez-Freire et al. (13) demonstrated that overexpression of ACE2 exerts protective effects on high BP and cardiac pathophysiology in SHRs.

Altogether, these studies suggest important roles of ACE2 in the cardiovascular system. This enzyme might be an important target in the treatment of cardiovascular diseases (21). For instance, the administration of XNT (1-[(2-dimethylamino)ethylamino]-4-(hydroxy-methyl)-7-[(4-methylphenyl)sulfonyloxy]-9H-xanthene-9-one), an ACE2 activator (22), promoted a decrease in BP, an improvement in cardiac function, and reversed myocardial and perivascular fibrosis in SHRs (14). Diminazene aceturate (DIZE-ACE2 activator) induced beneficial cardiovascular effects through the attenuation of cerebral infarction (23), prevention of pulmonary hypertension (24), enhancement of cardiac function (25), and modulation of the autonomic nervous system (24). However, there are no studies in the literature that demonstrate potential cardiovascular benefits of DIZE in association with physical exercise. Thus, the present study sought to determine the cardiovascular effects of DIZE in association with aerobic training in SHRs.

## Material and Methods

### Animals

All experiments were performed during 12 weeks using male SHRs (280–350 g). Rats were housed in a temperature-controlled room (22°-24°C) on a 12:12 light-dark cycle with free access to food and tap water. All experimental procedures were approved by the Institutional Animal Care and Use Committee of the Universidade Federal de Goiás (protocol #172/09) and were performed in strict accordance with the National Institutes of Health Guide for the Care and Use of Laboratory Animals.

### Experimental design and physical exercise

The SHRs were acclimatized for 2 weeks on a treadmill (EP-131, Insight, Brazil) prior to the 8 weeks of aerobic training (five times a week, lasting 60 min, at an intensity of 50–60% of maximum aerobic speed). During this period, 10 short sessions of workouts that lasted for 5–20 min/day (0% incline and speed of 5 to 12 m/min) were conducted.

After the acclimatization period, animals were subjected to progressively incremental exercise test. Speeds corresponding to 50 and 60% of the maximum speed were calculated and considered as training speed. The physical exercise began with a shorter duration and increased gradually over the weeks. At the beginning, the training load was equivalent to 50% of maximum speed during 2 weeks. In order to avoid adaptation, the load was gradually increased until 60% maximum speed was attained in 2 consecutive weeks. After 4 weeks of training, a new progressive maximal exercise test was carried out to establish new maximum aerobic speed for all animals. The physical exercise always began with 5 min of warm-up (5 m/min speed), followed by the endurance exercise and ending with 5 min of active recovery (5 m/min speed) (5). During the training period, the animals in the sedentary group (untrained animals) were placed on the treadmill to experience the same noise and environmental stress as the trained group.

In the last 15 days of the experiment, these groups were redistributed into four groups: i) sedentary SHRs with daily intragastric administration of 1 mg/kg DIZE (S+D1); ii) trained SHRs with daily intragastric administration of DIZE 1 mg/kg (T+D1); iii) sedentary SHRs with daily intragastric administration of vehicle (S+V; 0.15 M NaCl); and iv) trained SHRs with daily intragastric administration of vehicle (T+V).

### Surgical procedures, BP and HR recordings

After treatment, the animals were anesthetized with 2–3% halothane (Cristália Ltda., Brazil) and 100% O_2_ and polyethylene cannulas were implanted in the femoral artery to record the pulsatile blood pressure, mean arterial pressure (MAP), systolic blood pressure (SBP), diastolic blood pressure (DBP) and for intravenous drug administration. The cannulas were transfixed subcutaneously and externalized. Pentabiotic (200 mg/kg; Fort Dodge, Brazil) and 1.0 mg/kg flunixin (Chemitec, Brazil) were administered intramuscularly to prevent infections and pain, respectively. After the cannulation procedure, a bipolar electrode was implanted to record the electrocardiogram (ECG) and heart rate (HR) (26–28).

Twenty-four hours later, animals were subjected to baroreflex sensitivity test using 20 µg/kg, *iv* phenylephrine (α_1_-adrenergic agonist; Sigma Aldrich, USA). Intrinsic heart rate (IHR) was evaluated by double autonomic blockade (DBA) using 4 mg/kg atropine *iv* (Sigma Aldrich) and 2 mg/kg metoprolol *iv* (Sigma Aldrich).

### Morphometric analysis of the heart

At the end of experimental protocol, rats were anesthetized with 2–3% halothane and 100% O_2_ prior to decapitation. The hearts were removed and the ventricular chambers were separated and weighed. Left ventricular mass index (VMI) was calculated through the ratio between the left ventricular wet weight and tibia length.

### Data analyses

Data were reported as means±SE. Statistical analyses were done using GraphPad Prism software (v. 6; GraphPad Software, Inc., USA). The cardiovascular and morphometric parameters results were compared using parametric one-way ANOVA followed by the Fisher LSD *post hoc* test. P<0.05 was considered to be a significant difference.

## Results

### Running speed performance

The speed of the aerobic training increased in both trained groups (T+V: 26.3±0.3 to 41.2±0.6 and T+D1: 26.7±0.4 to 42.0±0.8 m/min, P<0.05). After 8 weeks of training, there were no significant differences in speed values between these groups.

### Recordings of cardiovascular parameters

**Figure 1:**
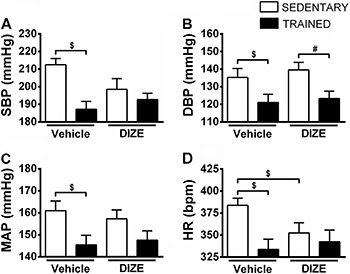
Means±SE of systolic blood pressure (SBP; *A*), diastolic blood pressure (DBP; *B*), mean arterial pressure (MAP; *C*) and heart rate (HR; *D*) of non-anesthetized sedentary spontaneously hypertensive rats (SHRs) that received intragastric administration of vehicle or diminazene aceturate (DIZE; 1 mg/kg) and trained SHRs that received intragastric administration of vehicle or DIZE. ^#,$^P<0.005 (ANOVA followed by the Fisher LSD *post hoc* test).

Exercise training promoted a decrease in SBP, DBP, and MAP (T+V: 187.2±4.4, 121.1±4.7, 143.2±4.3 mmHg, respectively, P<0.05) compared with sedentary SHRs (S+V: 212.5±3.6, 135.3±5.1, 161.0±4.3 mmHg, respectively; Figure 1). Exercise training in association with DIZE decreased DBP compared with sedentary SHRs treated with DIZE (T+D1: 123.3±4.1 *vs* S+D1: 139.5±4.4 mmHg; Figure 1B). The SBP and MAP values were not altered significantly between groups that were treated with DIZE (T+D1: 192.7±3.6, 147.6±4.3 *vs* S+D1: 198.5±6.1 and 157.3±4.0 mmHg, respectively; Figure 1A and C).

A decrease in HR was observed in the T+V (333.7±11.6 bpm) and in the S+D1 (352.4±11.6 bpm) as compared to S+V (383.8±8.0 bpm; P<0.05). Furthermore, the HR values of T+D1 group were not significantly lower compared to T+V and S+D1 groups (342.5±13.2 *vs* T: 333.7±11.6, and S+D1: 352.4±11.6 bpm, respectively, P>0.05; Figure 1D).

### Baroreflex sensitivity

**Figure 2:**
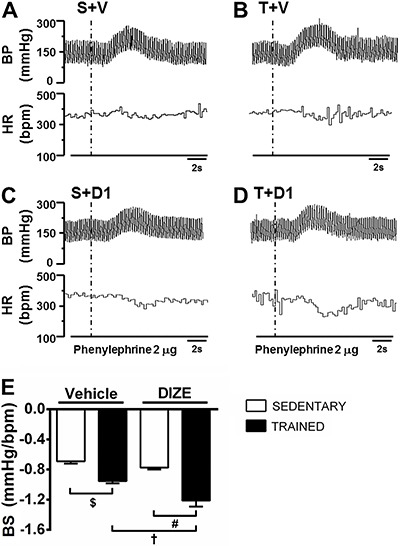
Representative traces of the cardiovascular responses induced by phenylephrine infusions in sedentary rats receiving vehicle (S+V; *A*), trained rats receiving vehicle (T+V; *B*), sedentary rats receiving diminazene aceturate (DIZE; 1 mg/kg) (S+D1; *C*) and trained rats receiving DIZE (T+D1; *D*). Baroreflex sensitivity (BS) is reported as the ratio between changes of heart rate (HR) and mean arterial pressure (MAP) induced by phenylephrine infusion (*E*). ^$,#,†^P<0.05 (ANOVA followed by the Fisher LSD *post hoc* test).

Figure 2 shows representative tracings of baroreﬂex activation by intravenous infusion of phenylephrine in sedentary and trained groups that were treated with vehicle (A and B, respectively) and DIZE (C and D, respectively).

As expected, training exercise improved the baroreflex sensitivity (T+V: −0.955±0.030 *vs* S+V: −0.693±0.030 Δ bmp/Δ mmHg; P<0.05). Interestingly, DIZE treatment potentiated this effect (T+V: −0.955±0.030 *vs* T+D1: −1.212±0.080 Δ bmp/Δ mmHg; P<0.05). The baroreflex sensitivity in both sedentary groups was not altered significantly (S+V: −0.693±0.030 *vs* S+D1: −0.775±0.024 Δ bmp/Δ mmHg; Figure 2E).

### Cardiovascular effects of double autonomic blockade

**Figure 3:**
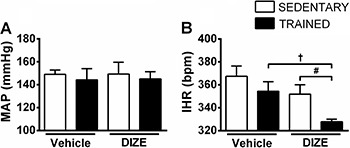
Means±SE of mean arterial pressure (MAP; *A*) and intrinsic heart rate (IHR; *B*) of sedentary and trained rats receiving vehicle or diminazene aceturate (DIZE) after double autonomic blockade. ^#,†^P<0.05 (ANOVA followed by the Fisher LSD *post hoc* test).

The double autonomic blockade significantly decreased the IHR of T+D1 group compared to the other groups (T+D1: 327.8±2.2 *vs* T+V: 354.4±8.3; S+D1: 351.8±8.3; S+V: 367.5±9.0 bpm, P<0.05; Figure 3A) without significant alteration in MAP (S+V: 149.0±3.8; T+V: 144.2±9.8; S+D1: 149.3±10.4; T+D1: 145.1±6.3 mmHg; Figure 3B).

### Morphometric analysis of the heart

**Figure 4:**
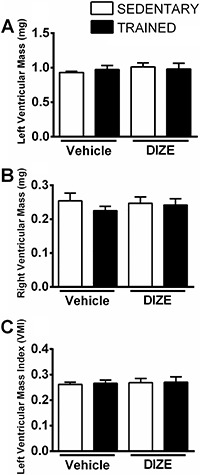
Means±SE of left ventricular mass (*A*), right ventricular mass (*B*), and left ventricular mass index (*C*) of sedentary and trained groups receiving vehicle or diminazene aceturate (DIZE).

No significant differences were observed in the left ventricular mass, right ventricular mass and left ventricular mass index among the groups (Figure 4).

## Discussion

Training exercise has been considered as a non-pharmacological and auxiliary method in the treatment of hypertension (3–5). In addition, previous studies have suggested a relationship between exercise and the RAS. The modulatory role of ACE2 on cardiovascular system (12–14,29) makes this enzyme an important therapeutic target. However, the impact of physical exercise in association with ACE2 activator on cardiovascular regulation remains unknown. The current study showed that association of training exercise and an ACE2 activator promoted: i) a decrease of resting HR, ii) a reduction of intrinsic bradycardia, and iii) an improvement of baroreflex sensitivity. These results strongly indicate that a combination of non-pharmacological and pharmacological therapies can enhance cardiovascular control in SHRs. Moreover, it seems that the increase in baroreflex sensitivity can lead to a reduction in BP variability.

Systematic exercise training as a non-pharmacological and auxiliary method in the treatment of cardiovascular diseases has been the focus of several studies (1,). A number of studies have demonstrated the beneficial effects of exercise on vascular reactivity (2,7), control of metabolic syndromes (2,6), attenuation of IRH (30) and ventricular hypertrophy (1,2). In the present study, training exercise promoted bradycardia, and systolic and diastolic hypotension.

Recent studies showed that the cardiovascular benefits induced by exercise are partially mediated by the central nervous system (5,31,32). Studies by Cruz et al. (31) showed that in the absence of peripheral chemoreceptors, the neuroplasticity induced by exercise in the paraventricular nucleus of the hypothalamus (PVN) pre-autonomic neurons was abolished. The authors also observed that the removal of the carotid body prevented exercise-induced resting bradycardia (31). Moreover, Stern et al. (33) demonstrated that sedentary SHRs have an increased excitability of PVN neurons. Moreover, these authors showed that exercise training reduces this neuronal excitation to levels similar of those observed in Wistar-Kyoto rats.

Exercise also induced important modifications in the peripheral vessels. Jordão et al. (34) demonstrated an increase in RNA expression of α-actin, elastin, and collagen in thoracic aorta, and an increase in wall volume and cross-sectional area of sedentary SHRs. In this study, training exercise restored all these parameters to normal levels.

In the present study, it was observed that exercise is efficient in promoting resting bradycardia. Similar results for resting bradycardia were also found by other authors (5,28). This resting bradycardia could occur through various factors including an improved tissue perfusion, and a decrease in total peripheral resistance by dilating agents such as bradykinin and nitric oxide (2,3,5,6). In addition, exercise training promotes a reduction in ANG II, an increase in Ang-(1-7) and subsequent decrease in sympathetic activity and HR (2,7,30).

The association of a non-pharmacological with a pharmacological treatment (DIZE) resulted in a significant reduction in DBP of SHRs as compared with sedentary SHRs. Like captopril, previous report showed that DIZE promoted hypotensive effects in the 2R1C model of hypertension (29). As mentioned before, DIZE is an activator of ACE2 (33), which in turn produces ANG-(1-7), mainly from ANG II (13). Biological activities of ANG-(1-7) include potentiation of RAS vasodilator axis, resulting in a pronounced hypotensive effect.

Although the association of exercise and DIZE potentiated a bradycardic effect, the hypotensive effect of DIZE remained unaltered by physical exercise. These data could be attributed to: i) type and intensity of exercise; ii) time of administration and DIZE dose (29). Other studies have demonstrated that chronic oral treatment with ANG-(1-7) (HPB-CD/ANG-(1-7)) promoted hypotensive effects in SHRs (3).

Rigatto et. al. (24) demonstrated that DIZE treatment re-establishes cardiac autonomic balance in monocrotaline-induced pulmonary hypertension (24). An orally active ANG-(1-7) in hydroxy-propyl-beta-cyclodextrin (HPB-CD) improved cardiovascular autonomic control assessed by spectral analysis of the HR and BP variabilities in SHRs (3). This result demonstrates that ANG-(1-7) can enhance the autonomic regulation of cardiac function. In the present study, DIZE treatment promoted a pronounced reduction of intrinsic HR in trained SHRs. These results indicate the involvement of RAS vasodilator axis in HR regulation. However, more experiments are still required to confirm this hypothesis.

The hypotensive and bradycardic effects of the exercise training could have involved RAS and other pathways. The ANG-(1-7) effects on HR are unclear. For instance, Braga et al. (35) demonstrated that ANG-(1-7) infusions promote sustained bradycardia in awake animals. In contrary, Santos et al. (36) demonstrated increase in HR in transgenic animals with superexpression of ANG-(1-7). Ferreira et al. (37), in turn, did not observe any effects of ANG-(1-7) on HR on isolated right atria. Our results demonstrated that chronic treatment with DIZE promoted bradycardia in sedentary SHRs.

A previous study (38) demonstrated that the binding of ANG-(1-7) to MAS receptor reduced the incidence and duration of reperfusion arrhythmias in isolated hearts. Furthermore, ANG-(1-7) enhanced the contractile function after an ischemic event. This effect suggests the involvement of prostaglandins and bradykinin (38). The expression of both ANG-(1-7) and its MAS receptor on sinoatrial node (37) reinforce the effectiveness of ANG-(1-7) in the attenuation of the arrhythmic reperfusion. The bradycardic effects of the exercise training could possibly involve an exercise-induced increase in ANG-(1-7) and MAS receptor expression on the sinoatrial node.

Recently, several studies have focused on the mechanisms underlining exercise training-induced hypotension. Gomes Filho et al. (39) observed that plasma Ang II is diminished in both Wistar and SHRs that were subjected to swimming training without changes in plasma ANG-(1-7). Moreover, a study by Ren et al. (19) demonstrated an exercise-induced attenuation of age-related increase of blood pressure. In addition, the expression of Ang II and ANG-(1-7) were reduced and increased, respectively, in the rostral ventrolateral medulla (RVLM) of trained SHRs. A decrease in oxidative stress in the RVLM of trained SHRs was also reported (19). It has been hypothesized that all these factors can contribute to the hypotensive effects of exercise training in SHRs. Recently, Silva Jr. et al. (20) reported that exercise training down-regulated the Ang II-AT1 axis in the renal artery without any alteration on the ANG-(1-7)-MAS axis of RAS.

Corroborating previous studies, we observed that baroreflex sensitivity was improved by exercise training, and interestingly, enhanced by the association of DIZE with exercise training. These results suggest that the modulation of RAS could improve reflex modulation of the autonomic system. Physical exercise is known to improve baroreflex sensitivity (5,9). In addition, intracerebroventricular infusion of ANG-(1-7) induced a significant increase in baroreflex sensitivity (40). However, to the best of our knowledge, no other study has demonstrated potentiation of baroreflex sensitivity in trained rats with DIZE treatment. Since the improvement of baroreflex sensitivity can elicit a reduction in BP variability, it is possible that this association can reduce BP variability-induced cardiac, vascular and kidney damage (8).

In summary, our findings showed that physical exercise in association with DIZE (activator of ACE2) elicited promising cardiovascular and autonomic effects. However, more studies are still required to clarify secondary effects of the combination of this ACE2 activator with exercise and the mechanism of cardiovascular effects.
